# BAMarray™: Java software for Bayesian analysis of variance for microarray data

**DOI:** 10.1186/1471-2105-7-59

**Published:** 2006-02-08

**Authors:** Hemant Ishwaran, J Sunil Rao, Udaya B Kogalur

**Affiliations:** 1 Department of Quantitative Health Sciences, Cleveland Clinic Foundation, 9500 Euclid Avenue, Cleveland OH 44195, USA; 2 Department of Statistics, Case Western Reserve University, 10900 Euclid Avenue, Cleveland OH 44106, USA; 3 Department of Epidemiology and Biostatistics, Case Western Reserve University, 10900 Euclid Avenue, Cleveland OH 44106, USA; 4 Ireland Comprehensive Cancer Center, Case Western Reserve University, 10900 Euclid Avenue, Cleveland OH 44106, USA; 5 Department of Statistics, Columbia University, 1255 Amsterdam Avenue, New York, NY 10027, USA

## Abstract

**Background:**

DNA microarrays open up a new horizon for studying the genetic determinants of disease. The high throughput nature of these arrays creates an enormous wealth of information, but also poses a challenge to data analysis. Inferential problems become even more pronounced as experimental designs used to collect data become more complex. An important example is *multigroup data *collected over different experimental groups, such as data collected from distinct stages of a disease process. We have developed a method specifically addressing these issues termed Bayesian ANOVA for microarrays (BAM). The BAM approach uses a special inferential regularization known as spike-and-slab shrinkage that provides an optimal balance between total false detections and total false non-detections. This translates into more reproducible differential calls. Spike and slab shrinkage is a form of regularization achieved by using information across all genes and groups simultaneously.

**Results:**

BAMarray™ is a graphically oriented Java-based software package that implements the BAM method for detecting differentially expressing genes in multigroup microarray experiments (up to 256 experimental groups can be analyzed). Drop-down menus allow the user to easily select between different models and to choose various run options. BAMarray™ can also be operated in a fully automated mode with preselected run options. Tuning parameters have been preset at theoretically optimal values freeing the user from such specifications. BAMarray™ provides estimates for gene differential effects and automatically estimates data adaptive, optimal cutoff values for classifying genes into biological patterns of differential activity across experimental groups. A graphical suite is a core feature of the product and includes diagnostic plots for assessing model assumptions and interactive plots that enable tracking of prespecified gene lists to study such things as biological pathway perturbations. The user can zoom in and lasso genes of interest that can then be saved for downstream analyses.

**Conclusion:**

BAMarray™ is user friendly platform independent software that effectively and efficiently implements the BAM methodology. Classifying patterns of differential activity is greatly facilitated by a data adaptive cutoff rule and a graphical suite. BAMarray™ is licensed software freely available to academic institutions. More information can be found at .

## Background

DNA microarray technology allows researchers to estimate the relative expression levels of thousands of genes simultaneously over different time points, different experimental conditions, or different tissue samples. It is the relevant abundance of the mRNA genetic product that provides surrogate information about the relative abundance of the cell's proteins. The differences in protein abundance are what characterize phenotypic differences between cells. Identifying such differences (even at the mRNA level) can lead to insight about biological processes and pathways that might be involved in a disease process as well as highlight new potential targets for diagnostic and therapeutic development. See [1-4] for more background on microarrays.

### Identifying signal in the presence of abundant noise

While potentially rich in information, microarray data pose a serious statistical challenge due to the sheer volume of information being processed [5]. It is the norm to see data collected on tens of thousands of genes from only a handful of samples. Data analysis is further complicated because of heterogeneity of gene-specific variances and correlation of gene expressions due to biological effect or technological artifact. Although many inferential questions are of interest, a common concern is of the detection of differentially expressing genes between experimental groups (e.g., between control samples and treatment samples, or between normal tissue samples and diseased tissue samples). Because of the large number of genes and tests involved, and because of the many inherent sources of noise in microarray data, the potential for Type-I errors or false detections is large. For two-group problems, a common strategy is to control the false discovery rate (FDR) using the method of [6] or empirical Bayes methods [7-9]. However, while these methods work well in controlling FDR, the price paid is often a conservativeness that leads to missing important genes [10]. Indeed, in two-group problems, the total number of misclassified genes can be derived in closed form assuming normally distributed data [11]. Such calculations suggest that when the fraction of truly differentially expressing genes is relatively low, total misclassification of differential effects will be large unless FDR is controlled at a high value, thus putting into question the value of such control.

### Multigroup data

The issues become more complex for *multigroup data *collected over different experimental groups, such as data collected from distinct stages of a disease process, or time course experiments in which microarrays are used to track gene expression profiles over time (the time points can be thought of as groups). The richness of such data lends itself to a myriad of potential questions and each question brings with it the thorny statistical problems associated with multiple testing. Because of this, most approaches start by simplifying multigroup hypotheses into a composite question that can be tested using a one-dimensional test statistic for each gene. While this is certainly convenient – for example, it makes it possible to apply standard error control methods such as the FDR – the strategy may not be optimal for several reasons. First, the underlying test statistic is likely to be fairly elementary, and thus highly variable because it will not be *regularized. *That is, the test is not likely to be constructed in a way that uses information across all genes and samples. Regularization is an important concept in microarray settings where sample sizes are small and the number of parameters are large (we will say more on this shortly). Secondly, composite statistics are seriously limited in the information they provide. Consider an *F*-test analysis involving contrasts for identifying specific patterns of differential expression across groups. For example, consider a gene that differentially expresses early on in a disease process, such as cancer, significantly affecting the biological milieu and making it possible for other genes to act, but then later vanishes. We call this a *hit-and-run *hypothesis. A contrast, or set of contrasts, looking for hit-and-run genes would simply provide what is equivalent to a *p-*value for rejecting the null hypothesis of no such pattern being present, but it would tell you very little about the likelihood of classifying a gene as having a hit-and-run pattern as apposed to some other pattern type.

### Rescaled spike and slab model selection and regularization

Recently Ishwaran and Rao [12], building upon work in [10], introduced a method for detecting differentially expressing genes between multiple groups termed Bayesian ANOVA for microarrays (BAM). This method recasts the statistical problem as a high dimensional model selection problem, and uses a specific Bayesian hierarchical model oriented towards adaptive shrinkage. By using model averaging, a way of accounting for model uncertainty, BAM provides gene effect estimates that are shrunken relative to standard least square estimates in which primarily only the non-differentially expressing gene effects are shrunken. This is a general phenomenon called *selective shrinkage *[12,13] that enables BAM to optimally balance total false detections (the total number of genes falsely identified as being differentially expressed) against total false non-detections (the total number of genes falsely identified as being non-differentially expressed). Selective shrinkage, theoretically, translates into more reproducible differential calls. BAM's ability to selectively shrink gene effects is an important form of regularization and is due to the use of a rescaled spike and slab model introduced by [13]. This model, in combination with a carefully selected continuous bimodal prior (also introduced in [13]), enables BAM to use data across all genes and all experimental groups to accurately estimate different levels of sparsity (the percentage of genes differentially expressing over a specific experimental group) and then to selectively shrink gene effects based on the estimated complexities. Equivalently, this procedure can be viewed as a penalization method in which each gene effect has a unique penalty term that is adaptively estimated from the data [12].

The BAM estimation procedure is fully automatic and is based on a Gibbs sampling algorithm. Not only are regularized differential gene effects estimated, but so is an *automatic data adaptive cutoff value *for determining which genes are differentially expressing. This cutoff value, for large enough sample sizes, has the theoretical property of delineating genes with true differential expression from those genes with no differential activity [12]. This is crucial, since determining an appropriate cutoff value is a critical aspect in searching for differential expression (whatever the method being used).

Another important feature in analyzing microarray data is the ability to systematically deal with heterogeneity of variances across genes and groups. Variance stabilization can lead to tremendous gains in power and is another important aspect of regularization. This issue was discussed in depth in [10,12] and Ishwaran and Papana (2005). BAMarray™ incorporates a nonparametric Classification and Regression Tree (CART) clustering algorithm described in Ishwaran and Papana (2005) to effectively deal with unequal variances. Of note is that the procedure does not artificially dampen or amplify group differences across genes for the sake of attaining variance stabilization.

### Illustrative example: tracking the genomic stagewise development of liver metastatic colon cancer

As preliminary illustration and motivation for BAMarray™, we look at expression data from a large microarray repository of colon cancer tissue samples comprising various stages of tumor progression. This data were obtained from Sanford Markowitz at the Ireland Cancer Center of Case Western Reserve University. All gene expression data were collected using high density 59K-on-one gene chips developed by EOS Biotechnology. These are Affymetrix-derived chips with proprietary probe sets. The high density of probe sets reflects known genes and ESTs (expressed sequence tags) as well as predicted exons.

**Figure 1:**
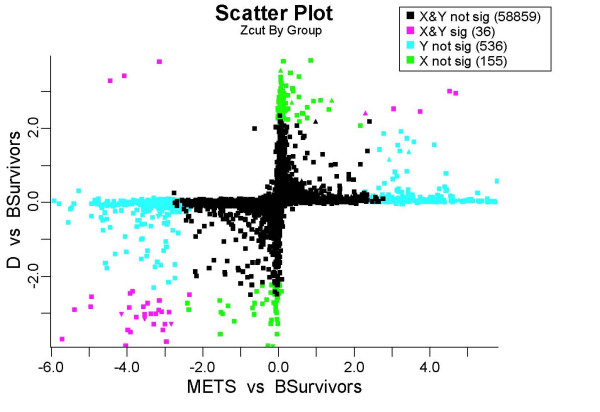
**Zcut values from the colon cancer analysis**. Vertical and horizontal axes are Zcut values measuring difference between D's versus BSurvivors and METS versus BSurvivors, respectively. Genes differentially expressing for both groups (magenta); D's but not METS (green); METS but not D's (blue); none (black). C versus BSurvivors differentially expressed genes are indicated by Δ (turning on) and ∇ (turning off).

Figure 1 shows a BAMarray™ analysis of the data using four distinct tissue samples: Duke's B, C, D and liver METS (the figure is produced as part of the graphical suite available in BAMarray™). The Duke's B samples represent Duke BSurvivors comprising patients still alive from the time of initial diagnosis. These represent an intermediate stage of cancer and form our control (baseline) group. We would like to track changes in gene expression across the stages of disease relative to this baseline. Duke's C samples represent a progressive worsening of the disease as the cancer begins to invade deeper into the colon wall and spread to nearby lymph nodes. The liver METS (METS) represent metastatic disease to the liver from the original primary tumor. The Duke's D samples represent the deposit left over in the colon after liver metastasis. Plotted in Figure 1 are BAM estimated gene differential effects (with respect to the BSurvivors) we call *Zcut values. *One can think of these as Bayesian test statistics. Figure 1 plots Zcut values for the METS and D's on the *x *and *y *axes respectively. Also overlayed on the plot are triangles for identifying genes turning off or on for stage C relative to the BSurvivors. The figure uses color to highlight stagewise gene effects of biologic interest. Points colored in magenta are genes with significant differential expression across the D's and METS. These are genes either being turned on or turned off relative to the BSurvivors. For example the small cluster of magenta triangles in the bottom-left quadrant indicate genes that turn off throughout the C, D and METS samples. Data points colored in green and blue indicate genes that are significant (in either direction) for only the stage D's but not the METS or only for the METS and not the stage D's, respectively. In particular, green points that hug the *y*-axis are those genes showing significant changes from BSurvivors to D's but whose METS expression resemble the BSurvivors. These are sometimes termed (early) *hit-and-run *genes because they differentially express early on in the disease progression, potentially significantly altering the biological milieu making it possible for other genes to act, but then later vanish in terms of biological effect. Note that statistical cutoffs used for identifying differentially expressing genes here have all been *adaptively estimated.*

**Figure 2:**
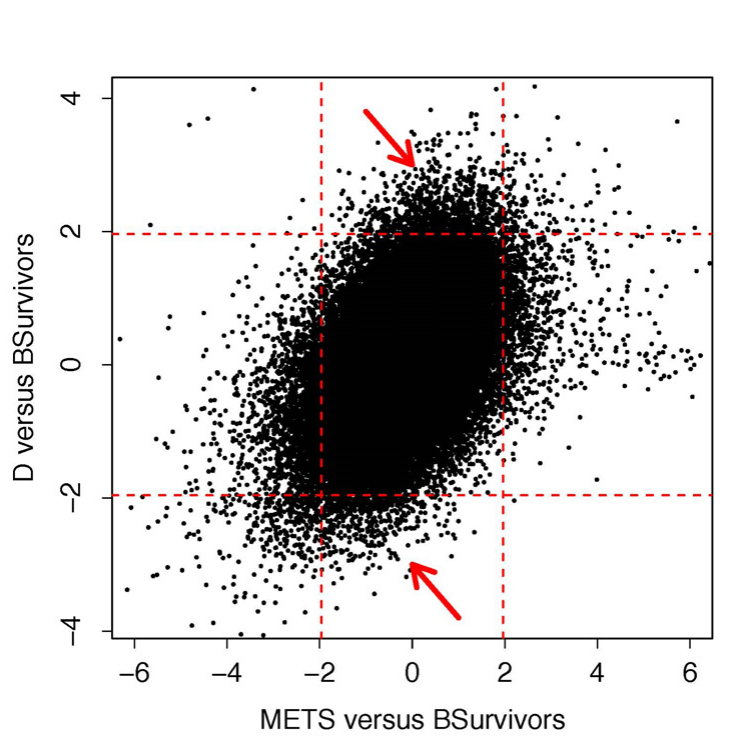
**Standard ANOVA ***Z***-test statistics (reproduced from [12])**. Arrows indicate quadrants containing potential hit-and-run genes using 95% confidence regions. Note the excess noise.

Standard least square test statistics (*Z*-tests) from a traditional ANOVA models provide a strikingly different plot. Figure 2 plots these values. Especially apparent is the ellipsoid nature of the figure. As was shown in [12], this is due to the high variability of the estimates and because of a regression to the mean effect caused by the correlation between the *Z*-statistics, in this case for METS versus BSurvivors and D versus BSurvivors genewise effect estimates. Regression to the mean inflates false detections and makes it more difficult to delineate signal from noise. Notice how difficult it is to identify any hit-and-run candidates. Early hit-and-run genes might be the ones in the quadrants indicated by the two arrows, but it is not so clear. This type of effect is strikingly absent in Figure 1 and clearly shows the benefits of shrinkage.

## Implementation

### Software architecture

BAMarray™ (Release 2.0) is a stand-alone platform independent desktop Java application. Solutions currently exist for the Mac OS X, Linux, and Windows XP operating systems. A native code C library is at the core of the product. This library implements the BAM algorithm and consists of several components including data pre-processing, data variance stabilizing transformations, and the Gibbs sampler. A Java graphical user interface surrounds the native code library and allows the user to interact with the library and conduct customized data analysis.

### Installing and uninstalling BAMarray™

BAMarray™ is available for download in the form of a self-extracting executable install package. Details can be found at . Users must register online in order to download the product. A 30-day evaluation license key will be automatically generated and emailed to the user upon registration (a full production license key will be emailed upon completing a signed license agreement). The user may then download the install package and execute the file according to the operating system specific protocol. The user completes the install by following the prompts generated by the package.

On first run, BAMarray™ will query the user for the license key. Once the key is verified, the product will present the user with the main console from which analysis can proceed.

Uninstalling BAMarray™ is as straightforward as the install process. An uninstall icon is produced during the install process in the product's home directory. Double-clicking on this icon will remove the product from the system. User modified data files will remain, but can be disposed of manually if so desired.

### Some key software features

1. BAMarray is a stand-alone platform independent desktop Java application. Solutions currently exist for the Mac OS X, Linux, and Windows XP operating systems.

2. Full multigroup analysis for up to 256 groups can be handled. Overlay multigroup plots (similar to Figure 1) are available for visualizing how genes are mapped to specific pattern types of differential expression across groups.

3. Graphical zoom-in and lassoing tools enable the user to interactively generate lists of differentially expressing genes.

4. Gene labels can be toggled on or off allowing genes of interest to be readily identified. Genes of interest (such as those making up a biological pathway of interest) can be highlighted using a selection list.

5. Gene lists of interest can be exported for further exploration.

6. Unequal variances across genes and groups are systematically handled by an automated pre-processing step.

## Results

### Note on normalization of microarray data

BAMarray™ assumes that the data to be analyzed has been suitably normalized (exact data formats and importing of data is discussed in subsequent sections). Normalization is simply the removal of systematic effects across samples that might bias inference. Two examples are batch effects in which samples were run, and dates that samples were extracted. Normalization can significantly affect microarray inferences [14]. The user is required to provide suitably normalized expression data to BAMarray™. Normalization procedures *are currently not provided *within the package, but a future release (3.0) will have this capability (see the Discussions section for details).

Normalization methods for two-color array data (such as cDNA arrays) are discussed in [15]. For Affymetrix oligonucleotide arrays, suitable options include the Affymetrix MAS 5.0 analysis suite [16] or robust multi-array analysis [17]. These, and other, procedures are available in Bioconductor [18].

### Data formats and importing data files

**Figure 3:**
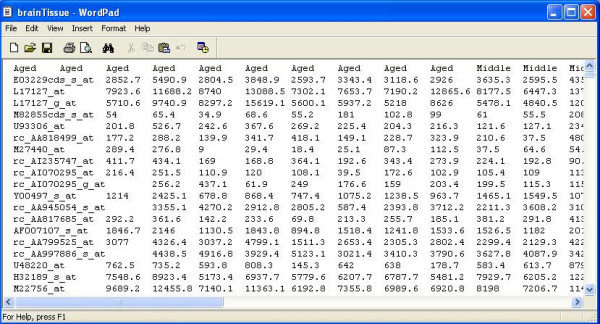
**Brain tissue data**. The first few lines of the brain tissue dataset (ASCII). Data comes bundled with the BAMarray™ install package.

**Figure 4:**
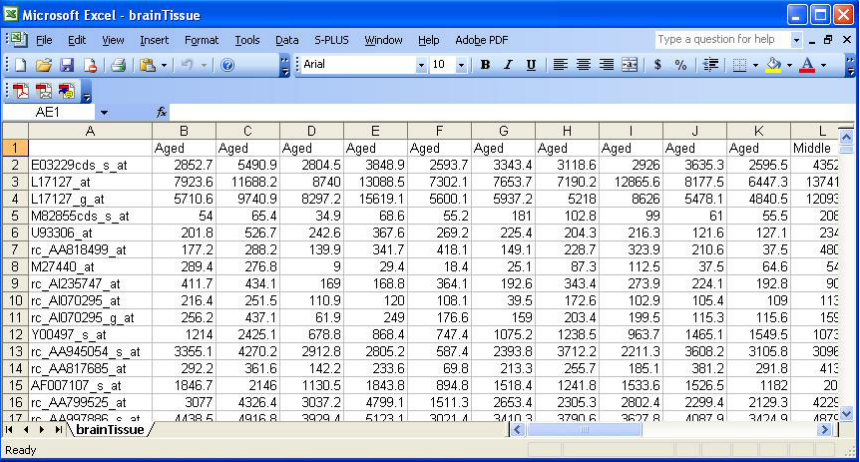
**Brain tissue data**. The first few lines of the brain tissue dataset (EXCEL). Data comes bundled with the BAMarray™ install package.

BAMarray™ supports microarray data in the form of an EXCEL spreadsheet or space-delimited text file (missing values are however not allowed). The first row of the file should contain class label information (i.e., the group label to which a particular sample belongs). This can be coded as letters and (or) integers. The first column of the dataset contains a gene label ID and is used for plotting and reporting purposes. Each subsequent entry following the first column is a suitably normalized gene expression measurement. There must be one row per gene, with each column representing a measurement for the sample identified in the first row. Figures 3 and 4 show the first few rows of an example data set (see below for more details) in text and EXCEL formats respectively.

### Illustrative example (bundled data)

The brain tissue dataset shown in Figures 3 and 4 (this will used for all illustrations henceforth) is a microarray experiment studying hippocampal aging and cognitive impairment. The goal of the experiment was to look for gene expression changes that track aging-dependent cognitive decline. Hippocampal CA1 tissue was collected from 4, 14, and 24 month old male Fischer rats after 7 days training on a water maze which included object memory task (see [19] for details). There were 10, 9 and 10 samples collected for the respective age groups. The age groups are labeled as Young, Middle, and Aged. The data are available at the National Center for Biotechnology Information (NCBI) Gene Expression Omnibus (GEO) data repository under series record accession number GSE854. This dataset comes pre-bundled with the default BAMarray™ installation. The default input directory (initialized when the user first starts the software) contains the brain tissue dataset.

### Importing data

**Figure 5:**
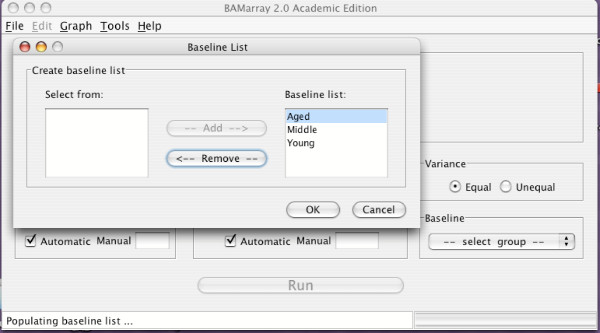
**Choosing groups to be used for the analysis**. The brain tissue dataset where all three groups (Young, Middle and Aged) have been chosen.

To open a microarray data set, click **New **under the **File **menu of the BAMarray™ main console and browse for the data. Once the file is found, click to highlight it, then click the **Open **button at the bottom of the Open File dialog box. Another dialog box will appear prompting for the groups to be used in the analysis. Groups can be added or removed by using the **Add **and **Remove **buttons respectively. Alternatively, for data with many groups, the user can select all groups (using SHIFT+END, or CTRL-A), or any subset (using SHIFT_PAGEUP, SHIFT_PAGEDN, SHIFT_ARROWUP, SHIFT_ARROWDN), instead of having to click on each group one at a time. All "standard" navigation keys can be used. Figure 5 shows the brain tissue dataset where all three groups have been chosen. After selecting the groups, click **OK**. The program will then read the data and notify the user of progress by way of the status bar at the bottom of the BAMarray™ main console. File name, number of groups, number of samples per group and total number of genes (probe sets) is provided in the file information panel on the main console.

### BAMarray™ run settings

**Figure 6:**
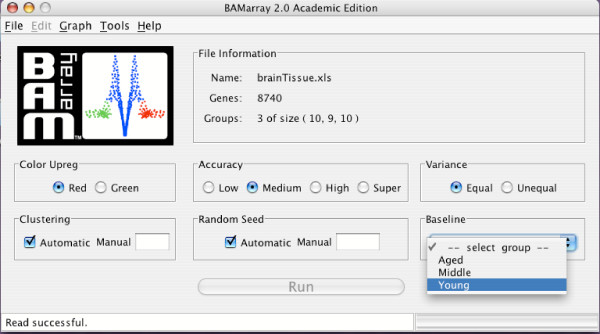
**Main console**. Main console. Group label "Young" is selected as the baseline for the analysis.

After the data is successfully read, several different run options can be selected from the main console (Figure 6). Many of these values are preset at well chosen default values and do not necessarily have to be adjusted (in fact, users are recommended not to adjust these values until they become familiar with the software and method).

The description of key run options are as follows :

(a) **Accuracy**: **Low, Medium, High **and **Super **settings correspond to the number of iterations for the Gibbs sampler. The Gibbs sampler is a Monte Carlo method for estimating parameter values of interest. The more iterations used (i.e., **Super **vs **Low**), the more accurate, but the longer the run time. For data exploration, a **Medium **setting will suffice. However, it is good practice to confirm results at the **High **or **Super **setting when possible.

(b) **Baseline: **This allows the user to define the baseline group for comparison purposes. The rationale for the baseline group is provided in [10,12]. It is typical to assign a control group or perhaps a normal or preliminary disease state as the baseline group. In our colon cancer example the BSurvivors represent the baseline, whereas in the brain tissue dataset the Young group serves as the baseline (see Figure 6). For time-course data the zero time point might be the most sensible baseline choice. Note that a **No Baseline **option is also available; details are provided later.

(c) **Clustering:**

**Automatic **and **Manual **settings. The default **Automatic **setting implements a variance stabilization and regularization step that systematically removes gene specific mean-variance trends. This is important to satisfy the constant variance assumption of the BAM model. The underlying method is based on a CART clustering approach and has the important feature that it does not alter the original signal to noise ratio of the data as seen with global transformation, such as logarithms (Ishwaran and Papana (2005)). Thus we recommend that users not pre-process their data and use the clustering procedure instead to provide variance stabilized transformed data. For advanced users a **Manual **option is used to pre-specify the number of clusters. One should carefully consult Ishwaran and Papana (2005) before experimenting with this option, however.

(d) **Variance: Equal **and **Unequal **settings. Expression values for genes are expected to have different variances (this is addressed by (c)). This option, however, indicates whether the variability of expression values differs over experimental group as well. The default **Equal **option implies equal variances across groups. Graphical diagnostic plots (to be discussed shortly) are provided for assessing if this assumption is met. For many applications, an equal variance model will be reasonable. For more details please consult [12] and Ishwaran and Papana (2005).

Clicking **Run **initiates the analysis. A status and progress bar at the bottom of the BAMarray™ main console indicate how long the Gibbs sampler will take and when the analysis has successfully completed.

### BAMarray™ graphics

The graphical suite becomes available once the Gibbs sampling step is completed. BAMarray™ graphics can be broadly grouped into two categories: *Data Plots *and *Inferential Plots.*

1. *Data Plots *are used to verify the assumption of equal variances. These include (i) cluster diagnostic plots, (ii) standard deviation plots, (iii) group mean plots, and (iv) V-plots (the last three are based on the transformed data).

2. *Inferential Plots *are based on estimated parameters from the model and are used for detecting differentially expressing genes. These include color enhanced shrinkage plots of Zcut values for identifying differentially expressing genes for a specific group. Also provided are multigroup Zcut scatter plots (similar to Figure 1 described earlier) for visualizing differentially expressing genes simultaneously over two or more groups.

### Data plots for assessing model assumptions: cluster diagnostic plots

**Figure 7:**
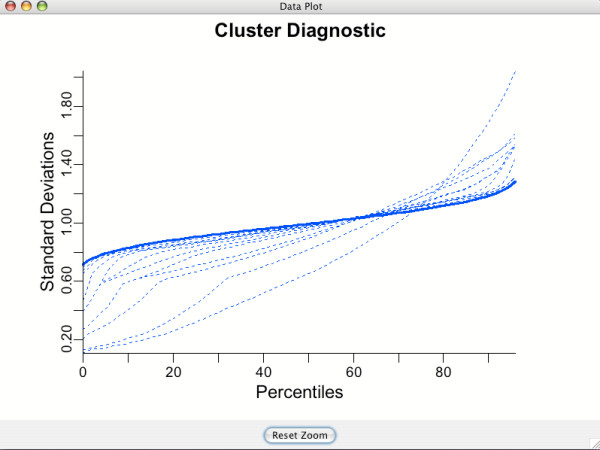
**Cluster diagnostic plot**. Used to assess the adequacy of the variance stabilization transformation (determined using a hybrid CART algorithm). As the number of clusters increases, the dashed lines (attempted transformations) get closer to the thick line (target distribution).

If the **Clustering **option is set to **Automatic**, BAMarray™ presents a cluster diagnostic plot for assessing the adequacy of the variance stabilization transformation. Figure 7 shows a "Cluster Diagnostic" plot. The solid blue line represents the percentiles for the theoretical target under a constant variance assumption. The dashed lines are values under the attempted transformations. As the number of clusters increases, the dashed lines will become closer to the solid line. See Ishwaran and Papana (2005) for more details.

### Data plots for assessing model assumptions: standard deviation, group mean difference and v-plots

**Figure 8:**
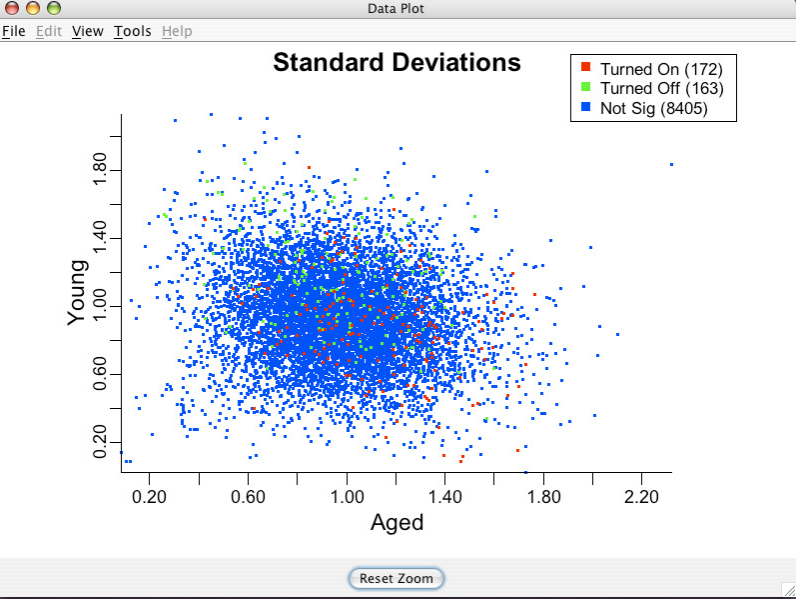
**Standard deviations for genes**. Standard deviations for each gene after the clustering transformation for brain tissue data. Used to assess the equal variance assumption. Green points indicate genes which are being turned off for Aged compared to Young. Red indicates genes being turned on for Aged compared to Young. Blue points are genes that are not differentially expressing.

Standard deviation and group mean difference plots can be examined to assess validity of the transformation. If an equal variance model has been approximately achieved, there should be no obvious trend visible in either of the above plots (i.e., they should look like random scatter). Figure 8 shows genewise standard deviations for groups Aged and Young on the transformed scales. Notice the lack of apparent structure and the relative tightness of the data points around the value (1.0,1.0) that is the target value.

**Figure 9:**
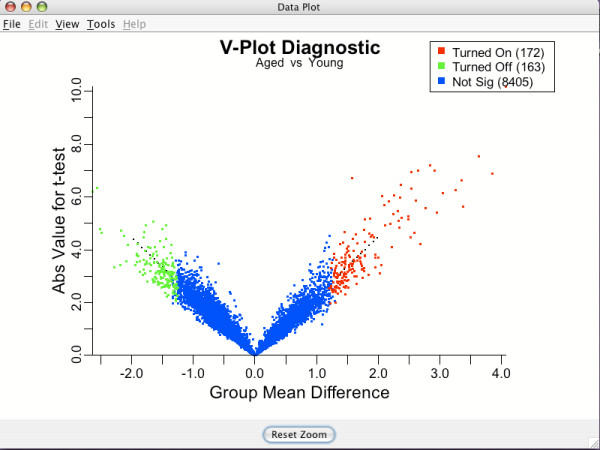
**V-plot**. V-plot of the Aged versus Young comparison in the brain tissue data. The tightness of the "V" is used to assess the equal variance assumption. Color defined as in Figure 8.

The V-plot [12] is another tool to assess adequacy of the equal variance transformation. If variances have stabilized to values near 1, then plotting the group mean difference for a gene versus the corresponding *t*-statistic in absolute values (both derived using transformed data) should give a plot with a line having constant slope. These theoretical lines (one in each direction of group mean differences) are overlayed as dotted black lines on the V-plot and the tightness around this line provides a graphical indication of the appropriateness of the transformation. See Figure 9.

### Inferential plots for detecting differentially expressing genes: shrinkage plots

**Figure 10:**
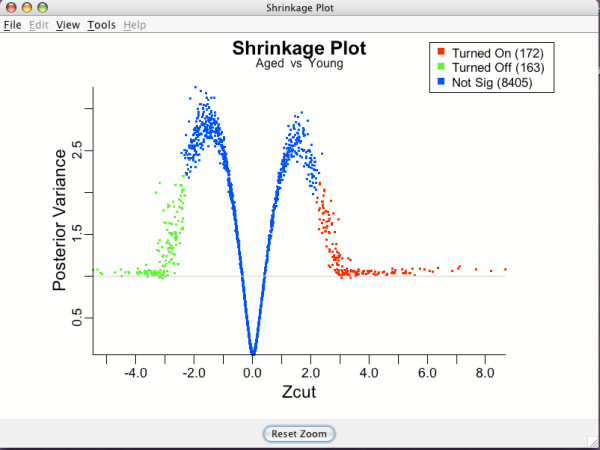
**Shrinkage plot**. Shrinkage plot for determining genes differentially expressing for the "Aged" group relative to the baseline group 'Young". Color defined as in Figure 8.

Shrinkage plots [10,12] are used to identify genes found to be differentially expressing (either up or down) relative to the baseline. Figure 10 is the shrinkage plot for the Zcut gene differential effects of the Age group from the brain tissue data (relative to the baseline group, Young). Green points indicate genes which are being turned off for the Aged group, whereas red indicates genes being turned on. Blue points are genes that are not differentially expressing.

The horizontal axis for the shrinkage plot are *Zcut *gene differential effects while the vertical axis are the corresponding posterior variances. Theoretical arguments show that genes that are truly differentially expressing will have posterior variances that coalesce to 1 on the far left and right sides of the plot. As the number of samples increases, eventually all of the truly differentially expressing genes will be found and none of the non-differentially expressing genes will be falsely detected [12]. BAMarray™ uses this principle to determine a data adaptive cutoff value.

### Inferential plots for detecting differentially expressing genes: multigroup scatterplots and zooming in

**Figure 11:**
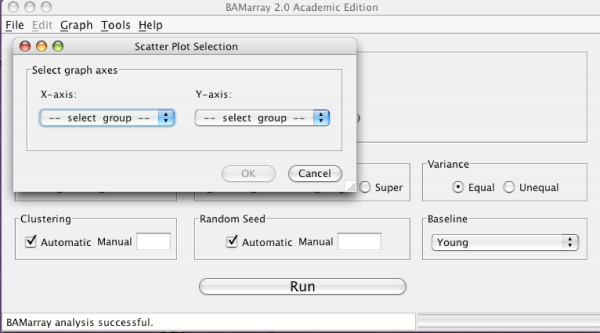
**Choosing axes for a multigroup plot**. Choosing which axes to use for a Zcut multigroup plot.

**Figure 12:**
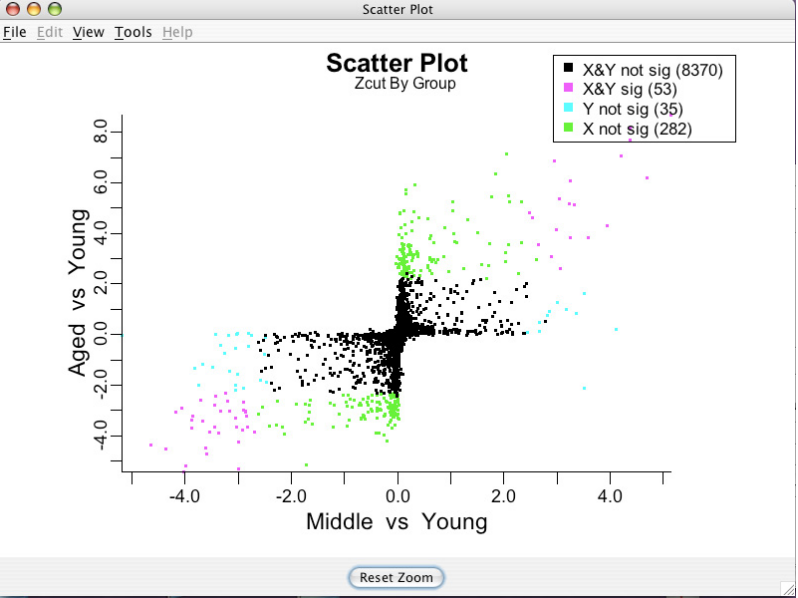
**Multigroup Zcut plot**. Zcut values by group colored by differential expression. Colors correspond to different expression profile types across groups for the brain tissue analysis.

Figure 12 presents a Zcut multigroup scatter plot. Figure 11 shows the dialog box used to generate the plot. The dialog box is used to select which Zcut values are plotted on the *x *and *y-*axes respectively. In the case of more than three groups the dialog box expands and includes an overlay option that allows an additional group's Zcut values to be superimposed on the plot. Triangles indicate genes expressing up or down for that group (recall Figure 1 where the overlay group was the Duke C's).

**Figure 13:**
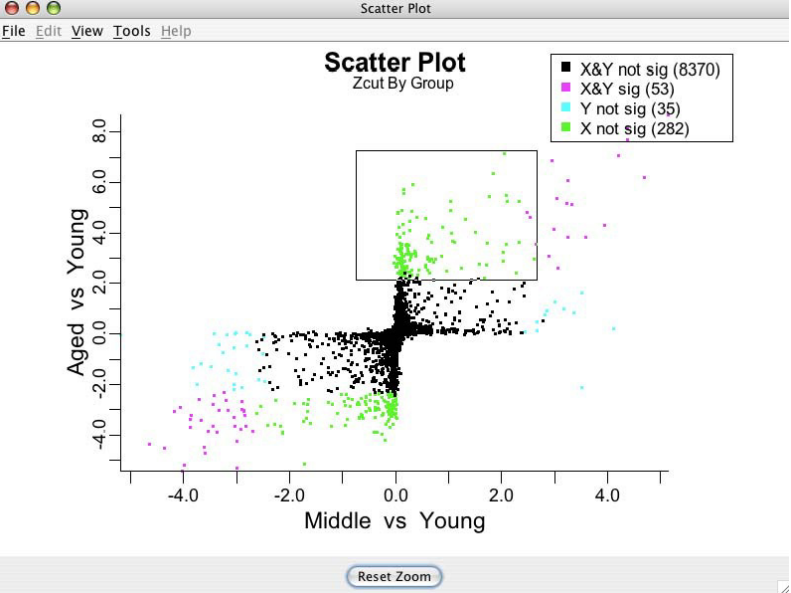
**Lassoing a region of interest**. Lassoed box of genes upregulated significantly for the Aged group but not the Middle aged group.

The legend in the top right corner of Figure 12 indicates how each gene is mapped to a particular pattern type of differential expression across the experimental groups. As in the shrinkage plot, the actual decision of whether a gene is significant and which group it belongs to is done automatically by BAMarray™. Different colors correspond to different expression profile types across groups. A gene could, for example, be significantly upregulated going from Young to Medium to Aged or down regulated going from Young to Medium but not from Medium to Aged. These patterns would correspond to the magenta color in the upper right hand quadrant of Figure 12 and the green data points hugging the negative *y-*axis respectively. Multigroup scatter plots are typically dense. It is often useful to zoom in on particular genes or particular gene expression patterns to improve clarity. Figure 13 shows the lassoing zoom-in feature available in BAMarray™. A lassoed box focused on only those genes that are significantly upregulated for the Aged group but not the Middle aged group is illustrated (the so-called *hit-and-run *genes).

**Figure 14:**
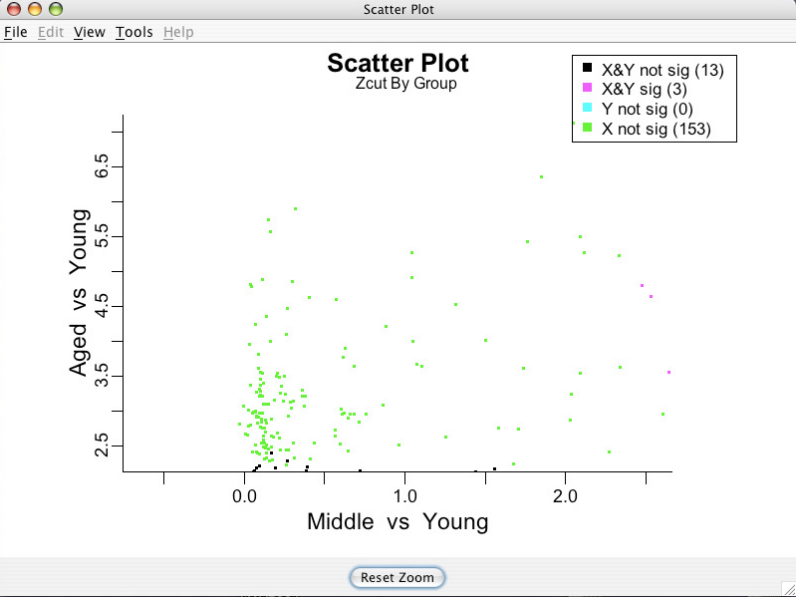
**Lassoed region**. Releasing the mouse lassoes the region of interest.

The lasso feature is activated by clicking and dragging the mouse cursor over a region of interest. Releasing the mouse button causes the plot to zoom in (see Figure 14). The user can repeatedly zoom in, even examining a single gene of interest if they choose. The original plot can always be restored by clicking the **Reset Zoom **button at the bottom of the plot. Lassoing is available on all data and inferential plots.

### Adding gene labels to plots and saving gene lists

**Figure 15:**
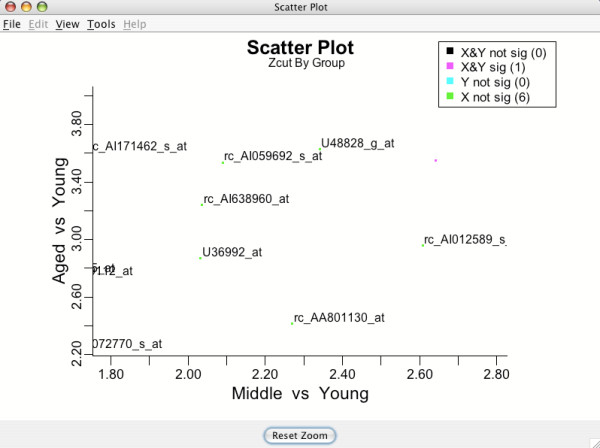
**Adding gene label IDs**. Gene labels can be added to almost any plot. Labels are toggled using the **View **menu item.

Gene labels can be toggled on or off on almost all BAMarary™ plots (Figure 15). To toggle labels, pull down the **View **menu item on the plot and click on the the desired gene subgroup. To overlay different subgroups, simply repeat the process by clicking on a new subgroup. A word of caution: If the zoom is reset, the original plot is restored but the labels will *still *be on. This can considerably slow the program down. However, gene labels for a particular subgroup can always be removed by going through the gene labeling process again and clicking the subgroup off.

Labeled (and unlabeled) genes can be saved as genes lists and output to a text file. To save all genes on the current plot pull down the **File **menu on the plot and click **Save Genes As...**. To add more genes to a previously saved list, simply use the **Append Genes **feature on the same menu column. For convenience BAMarray™ makes sure that appended lists of genes contain no duplicate gene labels. There is also a feature that allows the user to save all significant genes. This is found on the main console under the File Menu as **Save All Sig Genes...**. In particular, for users who are interested in a fully automated session, the procedure is:

1. Read in the data. Select the groups for the analysis and the baseline group. Click on **Run**.

2. When the analysis is complete, go to the main console under the File Menu and click on **Save All Sig Genes...**. This will save all significant genes and their classification values.

### Plotting options and using the gene tracking facility

BAMarray™ plots can be customized by pulling down the **Tools **menu item on any graph. This will highlight an **Options **command, which when activated, will open up a **Plot Options **window that highlights **Preferences**. Plotting label and character sizes can be adjusted here. Clicking the **Apply **button activates the desired changes. The default label and character sizes are 6 pt.

**Figure 16:**
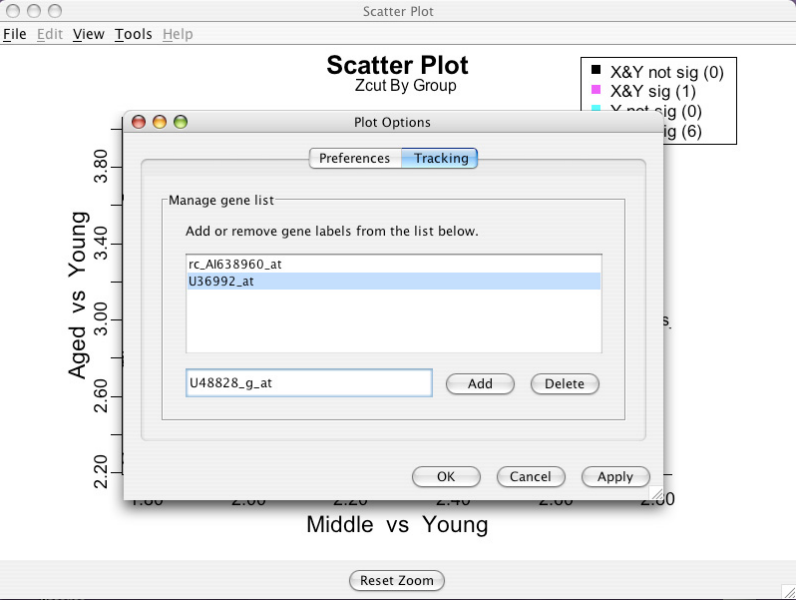
**Tracking specific genes of interest**. Genes can be tagged one at a time by sequentially entering gene names using the **Tracking **button in the **Plot Options **dialog box.

Highlighting the **Tracking **button in the **Plot Options **window (Figure 16) opens up a dialog box that allows the user to manually enter gene labels. Genes can be tagged one at a time by sequentially entering gene names and then clicking the **Add **button. The gene list of interest will then be updated and viewable in the display box above. Genes can be deleted from this list by highlighting those genes using the mouse and then clicking **Delete**. This feature can be used to track genes that make up a biological pathway of interest. Once a gene list has been produced, clicking **Apply **will cause all open plots to have genes in the gene list highlighted in boldface and their points enclosed in a dark circle. It can be handy sometimes to increase the plotting label size in order to clearly see the highlighted genes on all plots.

### More on assuming equal variances across groups

**Figure 17:**
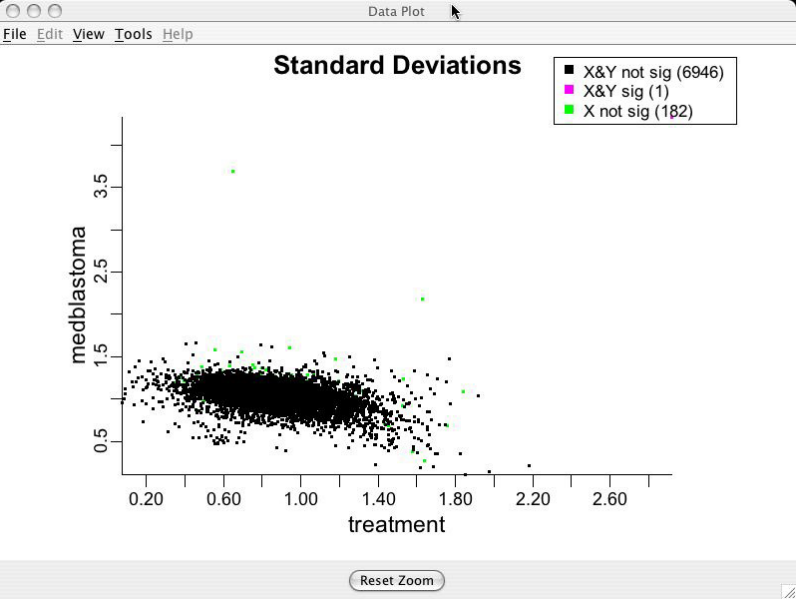
**Standard deviations assuming equal variances**. Data analyzed by assuming equal variances across groups. Note the *smear *effect that is typical of a violation of the equal variance assumption.

**Figure 18:**
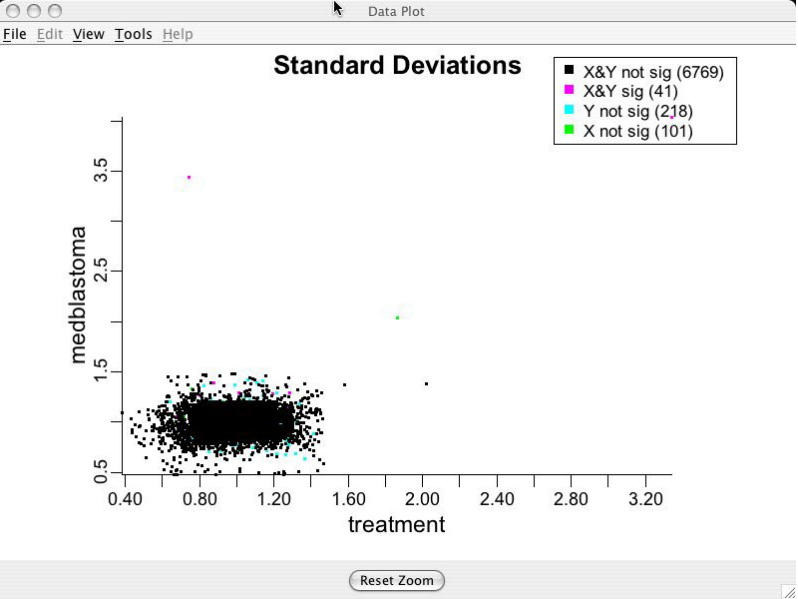
**Standard deviations assuming unequal variances**. Data analyzed by assuming variances are unequal across groups. Note the lack of smearing indicating a better fit.

As discussed earlier, BAMarray™ allows for group variances to be modeled as unequal. A tip-off that an unequal group variance transformation is required is shown in Figure 17 which shows a model with an unequal group variance structure fit under an equal variance assumption. The standard deviations plot shows a characteristic *smearing *effect indicating that the variance clustering algorithm was not able to achieve constant variance across genes. Contrast this with Figure 18 which uses the **Unequal **variance clustering option that allows for unequal group variances. Note how the smearing effect has all but vanished.

### The no baseline option

There are occasions when fitting a model with a **No Baseline **option is of interest. This option is accessible under the **Tools **menu on the main console under **Baseline Options**. Clicking on **No Baseline Selection **enables this feature for the session. No baseline means that each gene effect is being tested against a null value of zero (i.e. no detectable effect at all) rather than against a defined baseline group.

*Example: tracking tumor progression genes*. The colon cancer example presented earlier compared the various stages of colon cancer against the early onset BSurvivors group. In an informal sense, this analysis asks the question "what makes a good tumor go bad?" Another approach would be to identify genes that track the stagewise progression of metastatic colon cancer. This can be done by creating a new response variable, which measures the difference in gene expression between the successive stages of colon cancer, and then using a four-group analysis with no baseline in BAMarray™. The new response values would include modified stage C gene expression data created by comparing C measurements for a gene to some overall summary measurement for the corresponding BSurvivors gene. Similarly there would be a modified stage D measurement designed to measure difference from the D's to the C's. Finally we would have modified METS expression values recording differences between the METS and the D's. Each gene effect could then be tested against the null value of zero and statistical inference would reveal which genes have significant changes in gene expression as a function of stagewise progression of colon cancer.

## Discussion

The need for high quality software is rapidly growing in the area of genomic research. More powerful and elegant ways to store and analyze data are making mining the vast quantities of data we collect much more manageable and time efficient. Our main objective in producing BAMarray™ was to provide cutting edge statistical tools embedded within a sophisticated and easy-to-use graphical interface. Our goal was to free the user from as many subjective choices as possible and facilitate interactions with their data. While some knowledge of the underlying methodology is certainly useful, our main focus was to delineate the methodological ideas via simple, yet elegant, graphics that would make the software much more approachable for non-statisticians. Yet because the output from our software is stored in text format with clean and simple summary structures, it makes it possible for a more advanced statistical user to interface BAMarray™ with their own favorite products.

BAMarray™ has some key advantages which can be summarized and contrasted against other similar software. These include:

1. BAMarray is a stand-alone desktop Java application that interfaces with a native code C library. The software is highly portable and it is possible to create builds of the software for virtually any operating system. At this time, the software has solutions for the Mac OS X (10.3+), Linux, and the Windows XP operating systems.

2. BAMarray™ allows for a full multigroup analysis. This facilitates the searching for complex biological patterns such as the hit-and-run patterns of differential expression described earlier in this manuscript. Of course, many other applications are possible. For example, the very large number of experimental groups that can be analyzed (up to 256) facilitates studying expression changes in data settings where group labels could be tissue types collected from multiple regions within an organism (for human data this could be used for a genomic body map analysis for example).

3. BAMarray™ is nearly automatic in its usage. The user is freed from having to set and (or) choose tuning parameters, many of which can often affect the resulting conclusions [20]. Instead, we appeal to underlying theory to set tuning parameters at theoretically optimal values.

4. BAMarray™ importantly does not require a user-specified cutoff value for identifying significant genes. This is often the most difficult part of using a statistical software package. The choice of what is deemed significant is often arbitrary and dictated by available resources for follow-up analyses. Instead with BAMarray™, the cutoff values are set by appealing to the underlying theory via a novel shrinkage plot. From this plot, genes with posterior variances coalescing at a value of 1 are guaranteed to be truly differentially expressing with probability tending to 1.

5. Unequal variances across genes and (or) experimental groups is a common occurrence in multigroup studies. As described, global variance stabilizing transformations can be difficult to find and also unduly affect signal-to-noise ratios. BAMarray™ uses a sophisticated local variance stabilizing CART algorithm which does not suffer from the adverse properties of a global transformation. Importantly, this type of variance stabilization can also be used as a pre-processing step on its own. So even if a user would eventually like to analyze data in another package, variance stabilization can still be handled effectively in BAMarray™.

6. A no-baseline option in BAMarray™ allows for some non-standard experimental designs to be analyzed. This includes analyzing one-way ANOVA models (for example paired experimental designs), time course gene expression profiles, or perhaps tracking disease progression genes.

7. A suite of graphical tools are available in BAMarray™. These include diagnostic plots to check for the appropriateness of model assumptions and the adequacy of the pre-processing; zoom-in and lassoing features that allow the user to interactively generate lists of differentially expressing genes; toggling on or off of gene labels; and a gene tracker function that allows pre-specified lists of genes to be interactively tracked for differential expression across experimental groups.

8. Gene lists can exported to any software package that can read simple text files. A myriad of possibilities exist for follow-up analyses, but for most users, annotating gene lists would be of first importance. This can be done easily by importing significant gene lists from BAMarray™ into packages like GeneSpring, NetAffx™ or Bioconductor.

9. All figures can be saved as publication quality color graphics.

10. Analyses can be done at various levels of accuracy. This amounts to user-control over how many Gibbs sampling iterations are allowed. For most exploratory, first-wave analyses, a lower number of iterations would be sufficient. When conducting confirmatory analyses, a much larger number of iterations can be set.

### What's next?

Our illustrative example involving colon cancer showed how BAMarray™ can be used to track differentially expressing genes in multigroup experiments by statistically mapping genes to unique differential expression pattern types (for example, hit-and-run patterns). An important outcome of this is the ability to group genes by pattern type in order to find more focused underlying biology. We note, however, that higher order analyses like building molecular classifiers or survival outcome predictors can also benefit from this information. In fact, the very patterns that are found can be used in a special way to help build more powerful molecular models. This is work that we will report on shortly.

In addition to this work, the team continues to upgrade the software and a new release, Release 3.0, will soon be made available at . This major upgrade will contain some important enhancements to the product. For example, the capability to run BAMarray™ in an unattended Batch Mode initiated from a script file will be available. Batch Mode allows users to source BAMarray™ from any application that implements the use of operating system command driven script files. Writing custom designed scripts allow users to interface with different types of software, such as Bioconductor, and R, and could be used, for example, to automate the process of normalizing data. Release 3.0 will also have a Save Run feature allowing users to save the results of a run for later retrieval. A run that can take minutes to execute can be restored in only seconds using a Restore Run feature. Save Run can also be triggered in Batch Mode. This unique feature allows users to batch multiple jobs for later retrieval. Finally, Release 3.0 will allow users to populate a tracking list from gene labels found in an existing file. These and many more enhancements will be found in the next release of the product.

## Conclusion

BAMarray™ is user friendly Java-based software that effectively and efficiently implements the BAM methodology for analyzing expression data from multigroup experimental designs. The portability and flexibility of the product make it possible to rapidly adapt BAMarray™ to the highly dynamic field of genomic informatics and to modify the existing product to allow for seemless interface with other software and data mining tools as they become available.

## Availability and requirements

1. Project name: BAM

2. Project home page: 

3. Operating system(s): Windows XP, Linux, Mac OS X (10.3+).

4. Programming language: Java, C.

5. Other requirements: 512 MB RAM, 2.0 GHz Pentium 4 CPU, 200 MB free disk space on hard drive, Sun Java™ 2 Runtime Environment, Standard Edition (JRE) 1.4X. For Windows XP, installation must be done by users in the "Administrators" group or "Power Users" group only.

6. License: Academic and commercial license available from Technology Transfer Office at Case Western Reserve University. Details found at .

7. Any restrictions to use by non-academics: License needed.

## Authors' contributions

Hemant Ishwaran and J. Sunil Rao developed the theory and methodology underlying BAM. The colon cancer application evolved from research collaboration between J. Sunil Rao and Sanford Markowitz of the Ireland Cancer Center at Case Western Reserve University. Java software development, design and software engineering issues for BAMarray™ were handled by Udaya B. Kogalur. The native code C library was written by Hemant Ishwaran. All authors contributed to writing the manuscript.
